# Integrating cortical microtubules into plant gravity signaling: dynamics, tubulin phosphorylation, and growth regulation

**DOI:** 10.3389/fpls.2026.1970801

**Published:** 2026-10-08

**Authors:** Md Sifatul Islam, Sarah E. Wyatt

**Affiliations:** Molecular and Cellular Biology Program, Department of Environmental & Plant Biology, Ohio University, Athens, OH, United States

**Keywords:** cortical microtubule, cytoskeletal integration, gravitropism, gravity resistance, microgravity, PHS1, protein phosphorylation, α-tubulin

## Abstract

Plant gravity responses depend on coordinated signaling and growth-control mechanisms that convert directional and mechanical stimuli into differential cell expansion. Cortical microtubules are central to the conversion: they pattern cellulose synthase trajectories, set cell-wall anisotropy, and are remodeled in altered-gravity and tubulin-mutant systems. Yet the cytoskeletal regulatory pathway remains only loosely connected to the canonical, auxin-centered picture of how plants perceive and respond to gravity. Physiological, genetic, and phosphoproteomic evidence linking microtubules to gravitropism and gravity resistance, obtained under gravistimulation by reorientation at 1 g, hypergravity, and real or simulated microgravity, is organized into an explicit, layered framework. Three regulatory layers are proposed to act on cortical microtubule organization on distinct time scales: a slow transcriptional layer that adjusts the tubulin and regulator pool, an intermediate regulator-mediated layer of nucleation and severing, and a fast post-translational layer that modifies tubulin directly. Within the framework, phosphorylation of α-tubulin at threonine 349 (Thr349) by PROPYZAMIDE-HYPERSENSITIVE 1 (PHS1) is the best-characterized candidate for a molecular link between stress signaling and control of microtubule polymerization. Whole-seedling spaceflight phosphoproteomic data show reduced Thr349 phosphorylation across four α-tubulin isoforms (TUA1, TUA3, TUA4, and TUA6) and highlight Thr349 phosphorylation as a candidate post-translational regulator of cortical microtubule responses under spaceflight conditions. Future studies should test whether PHS1 controls Thr349 phosphorylation during gravity responses and identify the activating signal and opposing phosphatase. Resolving links with γ-tubulin-dependent nucleation, katanin-mediated severing, auxin transport, and cell-wall remodeling will explain how cortical microtubules shape gravity-directed growth.

## 1 Introduction

Gravity is a constant feature of the terrestrial environment, and plants have evolved to perceive both its direction and its mechanical load. Darwin’s observations on plant movement framed gravitropism as a directional response to a directional stimulus (Darwin and Darwin, 1880). Later physiology separated two related but distinct processes: gravitropism, the directional bending of an organ after a change in the gravity vector, and gravity resistance, the maintenance of organ form and growth against continuous gravitational loading (Hoson and Wakabayashi, 2015). Both processes engage intracellular signaling networks that include calcium fluxes (Plieth and Trewavas, 2002; Toyota et al., 2008), transient, spatially restricted accumulation of reactive oxygen species (Joo et al., 2001; Krieger et al., 2016), nitric oxide (Hu et al., 2005) signaling, MAP kinase signaling (Tran et al., 2026), and auxin redistribution (Friml et al., 2002).

How these components integrate has been difficult to resolve. The gravity-signaling network (Wyatt and Kiss, 2013) was described as a partially connected set of components, and Kruse and Wyatt (2022) later proposed a unified schematic centered on nitric oxide and reactive oxygen species. That synthesis clarified many canonical relationships, but the microtubule cytoskeleton was not its focus. The omission is notable because cortical microtubules reorient during gravitropic bending (Nick et al., 1990; Blancaflor and Hasenstein, 1993), remodel under altered gravity (Kato et al., 2022), and are altered in α-tubulin mutants with gravity-related phenotypes (Bao et al., 2001; Ishida et al., 2007; Matsumoto et al., 2010; Hoson et al., 2014). Recent studies have consolidated major advances in gravity sensing, including amyloplast sedimentation (Morita, 2010), LAZY protein repolarization (Chen et al., 2023; Nishimura et al., 2023), and the establishment of PIN-dependent auxin asymmetry (Friml et al., 2002). Their primary emphasis is gravity perception and early signal transduction, whereas the downstream contribution of cortical microtubules to cell-wall organization and differential growth remains less fully integrated.

Several reviews have addressed microtubules in tropisms (Bisgrove, 2008), cortical microtubules in gravity resistance (Hoson et al., 2010), and microtubule regulation during development and stress adaptation (Hsiao and Huang, 2023). The present review consolidates the altered-gravity, genetic, and phosphoproteomic evidence into a coherent framework for cytoskeletal integration, in which α-tubulin Thr349 phosphorylation emerges as a recurring candidate for a convergent node rather than a complete mechanism.

The review moves from cortical microtubule biology and the physiological evidence for gravity-responsive remodeling, through tubulin genetics and the transcriptional, regulator-mediated, and post-translational controls that shape array behavior, to a layered integration model in which Thr349 phosphorylation is examined as one candidate for a convergent node alongside the canonical auxin pathway (**Figure 1**). Throughout, direct experimental findings are separated from the inferences that connect them, and claims based on homology to non-plant systems are flagged rather than treated as direct plant evidence. Actin is considered only where it bears on the microtubule narrative.

**Figure 1:**
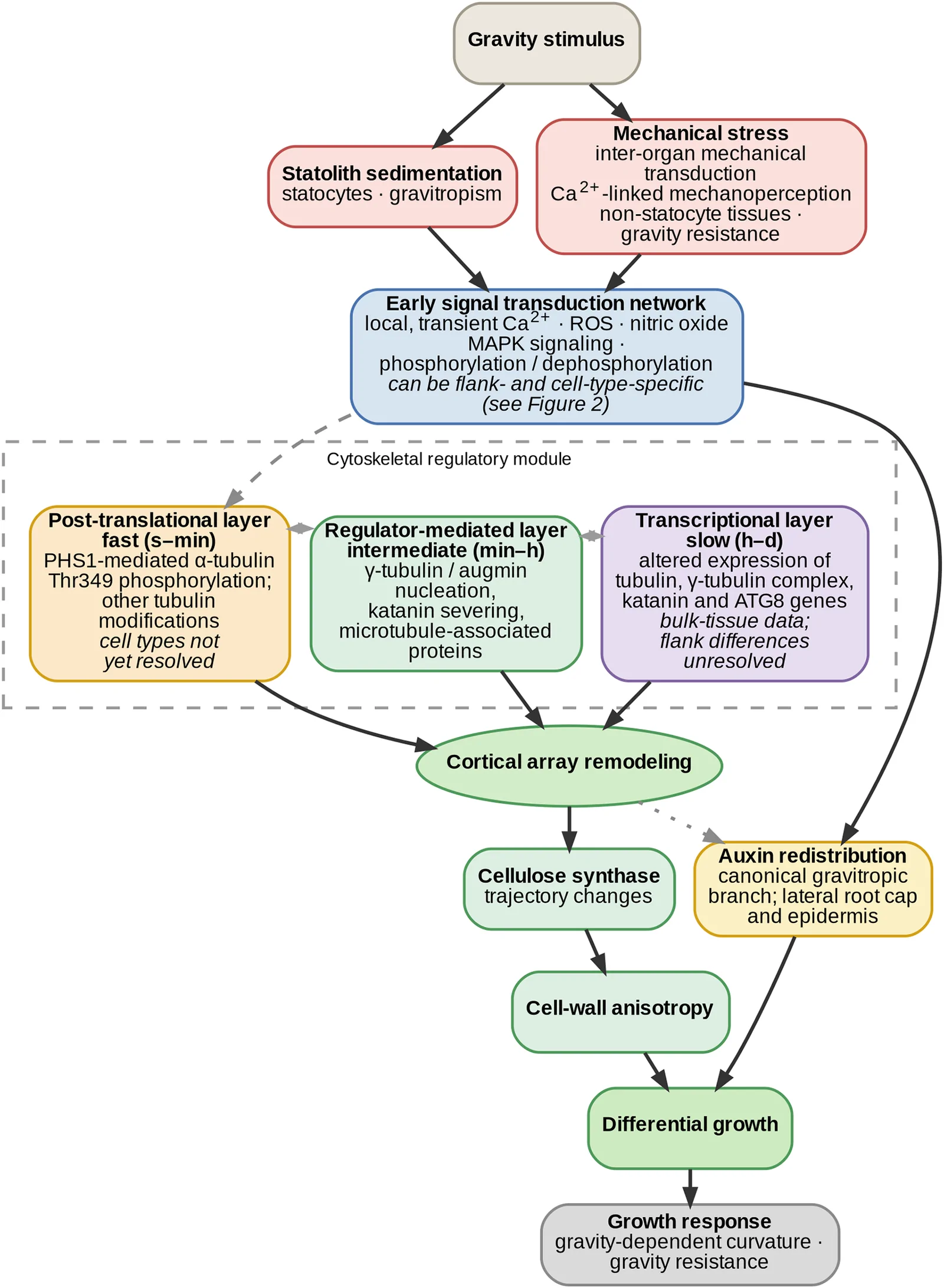
Cytoskeletal extension of plant gravity signaling. Read top to bottom as perception, transduction, cytoskeletal regulation, and growth. A gravity stimulus is perceived through two routes: statolith sedimentation in statocytes (gravitropism) and mechanical stress, including load transmitted between organs, sensed through Ca^2+^-linked mechanoperception in non-statocyte tissues (gravity resistance). Both feed an early transduction network of local, transient Ca^2+^, reactive oxygen species and nitric oxide signals, MAPK signaling, and phosphorylation/dephosphorylation, whose components can be flank- and cell-type-specific (**Figure 2**). The network drives the canonical auxin-LAZY-PIN branch, in which auxin is redistributed through the lateral root cap and epidermis (a route specific to roots), and is proposed to engage a cytoskeletal module of three layers: a fast post-translational layer (PHS1-mediated Thr349 phosphorylation and other tubulin modifications), an intermediate regulator-mediated layer (γ-tubulin/augmin nucleation, katanin severing, MAPs), and a slow transcriptional layer. These time scales describe the proposed regulatory framework and do not establish gravity-specific kinetics in every tissue. The cell types in which the post-translational layer operates are not yet resolved, and evidence for the transcriptional layer comes from bulk-tissue data in which flank differences are unresolved. The layers converge on cortical-array remodeling, which alters cellulose synthase trajectories, wall anisotropy, and differential growth alongside the auxin branch. Solid arrows denote established links; the dashed arrow from transduction to the cytoskeletal module denotes the central inference of the present review, supported but not yet directly demonstrated; the dotted arrow denotes predicted feedback onto auxin transport; dashed inter-layer connectors denote cross-layer interactions whose direction and hierarchy remain unresolved. The scheme summarizes regulatory logic, not spatial organization; *in vivo*, each component can differ in magnitude and sign between cell types and between the upper and lower flanks of a gravistimulated organ (**Figure 2**).

## 2 Cortical microtubule biology in plant cells

### 2.1 Architecture and dynamics

Microtubules are hollow cylindrical polymers approximately 25 nm in diameter, assembled from αβ-tubulin heterodimers. Heterodimers stack head-to-tail into protofilaments, and typically 13 protofilaments associate laterally to form the canonical microtubule. The polymer is intrinsically polar: the β-tubulin-exposed plus end is the primary site of dynamic growth and shrinkage, whereas the α-tubulin-exposed minus end is generally less dynamic and is often associated with nucleation. Both subunits bind GTP, but β-tubulin GTP is exchangeable and hydrolysable, while the α-tubulin GTP is buried at the intradimer interface and is not normally exchanged. Loss of the stabilizing GTP cap at the plus end can trigger catastrophe, a rapid switch from growth to shrinkage which may be followed by rescue. Dynamic instability is the biophysical substrate on which all microtubule regulators act (Nogales et al., 1999).

### 2.2 Plant-specific features

Plant microtubule organization differs from animal systems in several important ways. Plant cells lack centrosomes; new microtubules are nucleated at dispersed sites associated with the plasma membrane, the nuclear surface, and pre-existing cortical microtubules (Seltzer et al., 2007). γ-tubulin ring complexes provide nucleation activity, and the augmin complex contributes to microtubule-dependent microtubule nucleation in interphase arrays (Murata et al., 2005; Liu et al., 2014). Land plants also lack cytoplasmic dynein, leaving kinesins as the dominant microtubule-based motors. The most distinctive interphase feature is the cortical array, a quasi-two-dimensional network just beneath the plasma membrane that is central to morphogenesis because it helps orient cellulose deposition into the wall.

### 2.3 The microtubule-cellulose axis

Cortical microtubules and cellulose synthesis are functionally coupled. Cellulose synthase complexes (CSCs) move in the plasma membrane along trajectories aligned with underlying cortical microtubules (Paredez et al., 2006), and CELLULOSE SYNTHASE INTERACTIVE 1 (CSI1/POM2) provides a physical and functional link between CSCs and cortical microtubules (Li et al., 2012). Cellulose microfibril alignment is a major determinant of cell-wall anisotropy and therefore influences the direction of turgor-driven expansion (Baskin, 2005). Cortical microtubules contribute to directional growth by guiding cellulose synthase trajectories and influencing the orientation of newly deposited cellulose microfibrils (Paredez et al., 2006). Disruption of array organization by drugs, mutations, or stress-induced remodeling can alter CSC trajectories, cellulose deposition, and cell shape. Cortical microtubule arrays help guide cellulose synthase trajectories and cellulose microfibril orientation, thereby linking microtubule reorientation to cell-wall anisotropy and directional growth during gravitropism.

## 3 Physiological evidence for microtubule responses to gravity

Three experimental conditions are distinguished throughout this review. Gravistimulation refers to reorientation of an organ relative to the gravity vector at 1 g, for example by rotating a plate or pot, which changes the direction of the stimulus but not its magnitude. Hypergravity refers to centrifugation at forces above 1 g, which increases mechanical loading (for example, 300 g; Soga et al., 2006). Microgravity refers to near-weightlessness during spaceflight, for example aboard the International Space Station (ISS) (Hoson et al., 2014; Kato et al., 2022). Clinorotation and random positioning are ground-based approaches that simulate microgravity by continuously reorienting the plant (for example, three-dimensional clinorotation; Yemets et al., 2024), but they do not remove gravitational loading. Responses obtained under these conditions are not interchangeable and are identified by condition (**Table 1**).

**Table 1:** Spaceflight and altered-gravity experiments examining cortical microtubule responses in plants.

Reference	Gravity condition/facility	Species/tissue	Cytoskeletal observation	Molecular finding
(Soga et al., 2006)	Hypergravity (centrifuge), 300 g	*Vigna angularis* epicotyls	Transverse > longitudinal shift; suppressed by La^3+^, Gd^3+^	Reorientation scales with log of gravity magnitude
(Soga et al., 2008)	Hypergravity (centrifuge), 300 g	*Vigna angularis* epicotyls	Reorientation with transient γ-tubulin complex response	*VaTUG* and *VaGCP3* up; blocked by Ca^2+^-channel blockers
(Soga et al., 2009)	Hypergravity (centrifuge), 300 g	*Vigna angularis* epicotyls	Reorientation context; katanin examined	*VaKTN1* transcript increased transiently
(Matsumoto et al., 2010)	Hypergravity (centrifuge), 300 g	Arabidopsis hypocotyls; *tua3*, *tua4*, *tua6*	Twisting intensified; longitudinal fraction elevated	tua6 strongest; La^3+^, Gd^3+^ suppress effects
(Hoson et al., 2014)	Microgravity, ISS spaceflight, JAXA/Kibo	Arabidopsis inflorescences; WT and tua6	Elongation stimulated 10-45% under microgravity; tua6 more responsive	Cell-wall extensibility increased
(Kruse et al., 2020)	Microgravity, ISS spaceflight, BRIC-20/GLDS-38	Arabidopsis seedlings	Phosphoproteomic changes in cytoskeletal and wall proteins	Thr349 phosphorylation reduced under spaceflight conditions; CESA1 altered
(Kato et al., 2022)	Microgravity, ISS, Kibo Resist Tubule	Arabidopsis hypocotyls, inflorescence	Reorientation suppressed; cell elongation stimulated	Many tubulin genes downregulated; MAP responses variable
(Yemets et al., 2024)	Simulated microgravity, 3-D clinorotation	Arabidopsis roots	Microtubule disorganization; autophagosomes peak at day 6	*AtTUA2/3*, *AtTUB2/3*, *AtATG8b/f/i* upregulated

### 3.1 Cortical microtubule reorientation during gravitropic bending

Early immunofluorescence studies in maize coleoptiles, sunflower hypocotyls (Nick et al., 1990), and maize roots (Blancaflor and Hasenstein, 1993) showed that cortical microtubules can reorient during photo- and gravitropic curvature. Microtubule orientation is spatially associated with asymmetric growth, although the precise relationship varies with tissue, species, and developmental context. Pharmacological disruption of cortical arrays with oryzalin (Go et al., 2021) or propyzamide (Nick et al., 1991) impaired normal curvature, supporting a functional role for intact arrays rather than a purely correlative association. Taken together, available evidence supports a working model in which cortical microtubules function mainly during the growth-response phase of gravitropism, downstream of gravity perception and auxin redistribution (Friml et al., 2002; Ottenschläger et al., 2003), by influencing cellulose synthase trajectories, cell-wall organization, and differential cell expansion (Paredez et al., 2006).

### 3.2 Gravity resistance and the cell-wall framework

Gravity resistance refers to the cellular and tissue-level mechanisms by which plants maintain organ form under constant gravitational load. The framework (Hoson and Wakabayashi, 2015) emphasizes mechanoreception at the plasma membrane and downstream cell-wall reinforcement. Genetic evidence implicates MCA1 and MCA2 in the Arabidopsis hypocotyl response to hypergravity (Hattori et al., 2020). Cortical microtubules are central to the framework. In azuki bean (*Vigna angularis*) epicotyls, hypergravity at 300 g decreased the proportion of cells with transverse cortical microtubules and increased the proportion with longitudinal arrays; the response scaled with the logarithm of gravitational force, appeared within 0.5 h, and was suppressed by lanthanum and gadolinium (Soga et al., 2006). Under similar conditions, transcripts of γ-tubulin complex genes (Soga et al., 2008) and katanin p60 (Soga et al., 2009) increased transiently. Lanthanum and gadolinium also suppressed these transcriptional responses, supporting the involvement of mechanosensitive, calcium-permeable channels in early hypergravity signaling (Soga et al., 2008, 2009). Together, the results indicate that mechanical loading engages calcium-linked signaling, cortical microtubule reorientation, and gene-expression programs involved in microtubule reorganization.

### 3.3 Mechanical stress as a patterning input

Cortical microtubules align with predicted directions of maximal tensile stress in the Arabidopsis shoot apical meristem, establishing mechanical stress as a direct patterning input for array organization (Hamant et al., 2008). Because gravitational loading generates stress patterns that are transmitted across tissues and between organs, the framework predicts that microtubules in non-statocyte cells can respond to gravity-related mechanical stress independently of classical statolith-mediated perception, and that the responding cell need not be the cell in which the stress originates. Mechanical stress therefore provides a second route, alongside statocyte-mediated gravitropic signaling, by which gravity can engage the cortical microtubule cytoskeleton, a distinction that matters when asking where a given molecular response operates.

### 3.4 Spaceflight and simulated microgravity

Spaceflight and ground-based simulated microgravity provide complementary tests of microtubule responses to reduced gravitational loading (**Table 1**). In Arabidopsis inflorescence stems grown aboard the International Space Station (ISS), microgravity stimulated stem elongation by 10-45% relative to on-orbit 1 g controls, and the stimulation tended to be greater in the *tua6* α-tubulin mutant than in wild-type Columbia (Hoson et al., 2014). Cell-wall extensibility in elongating regions increased under microgravity, suggesting that removing gravity partly relieves growth constraints associated with gravity resistance.

In the Resist Tubule experiment on the Kibo module of ISS, epidermal cells in Arabidopsis hypocotyls were longer under microgravity than under on-orbit 1 g, and the normal transverse-to-longitudinal reorientation of cortical microtubules was suppressed (Kato et al., 2022). That study also reported that many α- and β-tubulin genes were downregulated under microgravity in apical inflorescence tissue, while microtubule-associated protein (MAP) gene responses were variable. Integrated transcriptomic and phosphoproteomic analysis of Arabidopsis seedlings from the BRIC-20/GLDS-38 spaceflight dataset identified reduced phosphorylation at Thr349 in TUA1, TUA3, TUA4, and TUA6 under spaceflight conditions, with small, consistent negative log2 fold changes relative to ground controls, and additionally identified altered phosphorylation of CELLULOSE SYNTHASE 1, linking cytoskeletal and cell-wall regulatory layers in the same dataset (Kruse et al., 2020). Because PHS1-mediated Thr349 phosphorylation impaired α-tubulin incorporation and is associated with stress-induced cortical microtubule depolymerization, reduced phosphorylation under spaceflight conditions is interpreted as a potential microtubule-stabilizing response. In the BRIC-20 dataset, Thr349-containing phosphopeptides showed a modest decrease under spaceflight conditions, with log2 fold changes of approximately -0.24 to -0.26 (Kruse et al., 2020). Isoform-specific interpretation is limited because TUA1, TUA4, and TUA6 share the same Thr349-containing tryptic peptide. Therefore, the phosphoproteomic signal cannot distinguish which of these three isoforms contributed to the observed decrease. TUA3 was represented by a distinct peptide and showed a similar reduction (≈ -0.26). α-Tubulin transcript levels did not show a corresponding decrease, suggesting that the change in Thr349 phosphorylation cannot be explained simply by transcriptional downregulation. Because the BRIC-20 analysis used whole seedlings, these values are organism-level averages; they do not show in which tissues the reduction occurred and could mask larger changes of opposite sign in different cell types or flanks (Section 3.5). A three-dimensional clinorotation study subsequently reported maximal autophagosome formation in Arabidopsis root cells after six days of simulated microgravity, accompanied by microtubule disorganization and changes in root morphology with upregulation of *AtTUA2*, *AtTUA3*, *AtTUB2*, *AtTUB3*, and the *ATG8* genes *AtATG8b*, *AtATG8f*, and *AtATG8i* (Yemets et al., 2024). Because these datasets differ in platform, tissue, and time scale, their transcriptional responses do not converge on a single “altered gravity” signature (Section 5.1).

### 3.5 Cell-type and positional specificity

A root or stem is a cylinder of concentric tissues, and after gravistimulation, cells at different radial and azimuthal positions do not respond in the same way. In Arabidopsis roots, gravistimulation increased Ca^2+^ spikes in the root cap, particularly on the lower side of the lateral root cap and most markedly within the first 15 min after reorientation (Zhao et al., 2022); the frequency of lower-side spikes then declined (Li et al., 2024). Using a different protocol, in which roots held horizontally for 7 min were then rotated by 180°, imaging with the FRET-based Ca^2+^ sensor YC3.6 revealed opposite responses in the root epidermis: cytosolic Ca^2+^ decreased on the upper flank from about 3 min and increased on the lower flank from about 4 min after the reversal (Monshausen et al., 2011). Auxin response became higher on the lower side of the root within minutes of reorientation, the upper/lower difference in epidermal and lateral root cap cells was greatest at about 2 h, and the asymmetry was lost as the root tip approached 40°from the horizontal (Band et al., 2012). Tissue-targeted expression showed that auxin transport through the lateral root cap to elongating epidermal cells, and the auxin response of those epidermal cells, are required for root gravitropism (Swarup et al., 2005). Reactive oxygen species were higher on the lower side of the distal elongation zone epidermis 1 to 2 h after reorientation, and the asymmetry dissipated after a further 2 h (Krieger et al., 2016); in maize roots, a transient increase was reported in the endodermis on one flank (Joo et al., 2001). Cortical microtubule responses are also position-dependent. During photo- and gravitropic curvature of maize coleoptiles and sunflower hypocotyls, microtubules at the outer epidermal wall changed from transverse to longitudinal orientation on the slower-growing flank, whereas transverse orientation was maintained on the faster-growing flank (Nick et al., 1990). In gravistimulated azuki bean epicotyls, the proportion of epidermal cells with longitudinal microtubules increased on the upper flank and the proportion with transverse microtubules increased on the lower flank; these changes did not occur when the epicotyls were kept straight and were inhibited by the auxin-transport inhibitor naphthylphthalamic acid (Ikushima and Shimmen, 2005). In the Arabidopsis root apex, microtubule orientation and its responsiveness to suppressed cell expansion differ among developmental zones and among faces of the same cell (Panteris et al., 2013). In maize roots, however, differential growth of cortical cells during gravitropism was reported to be independent of the microtubular cytoskeleton (Baluska et al., 1996), so the contribution of microtubules may differ between tissues and organs. Consequently, a signal that increases on one flank and decreases on the other can appear unchanged, or only modestly changed, when whole organs or seedlings are averaged. The gravistimulation studies cited above compared only cells near the side facing the gravity vector and cells near the opposite side; **Figure 2B** is a hypothetical illustration of how such responses could be tested cell by cell between these positions. For most readouts, including cortical microtubule orientation and Thr349 phosphorylation in Arabidopsis roots, such resolution remains limited or absent.

**Figure 2:**
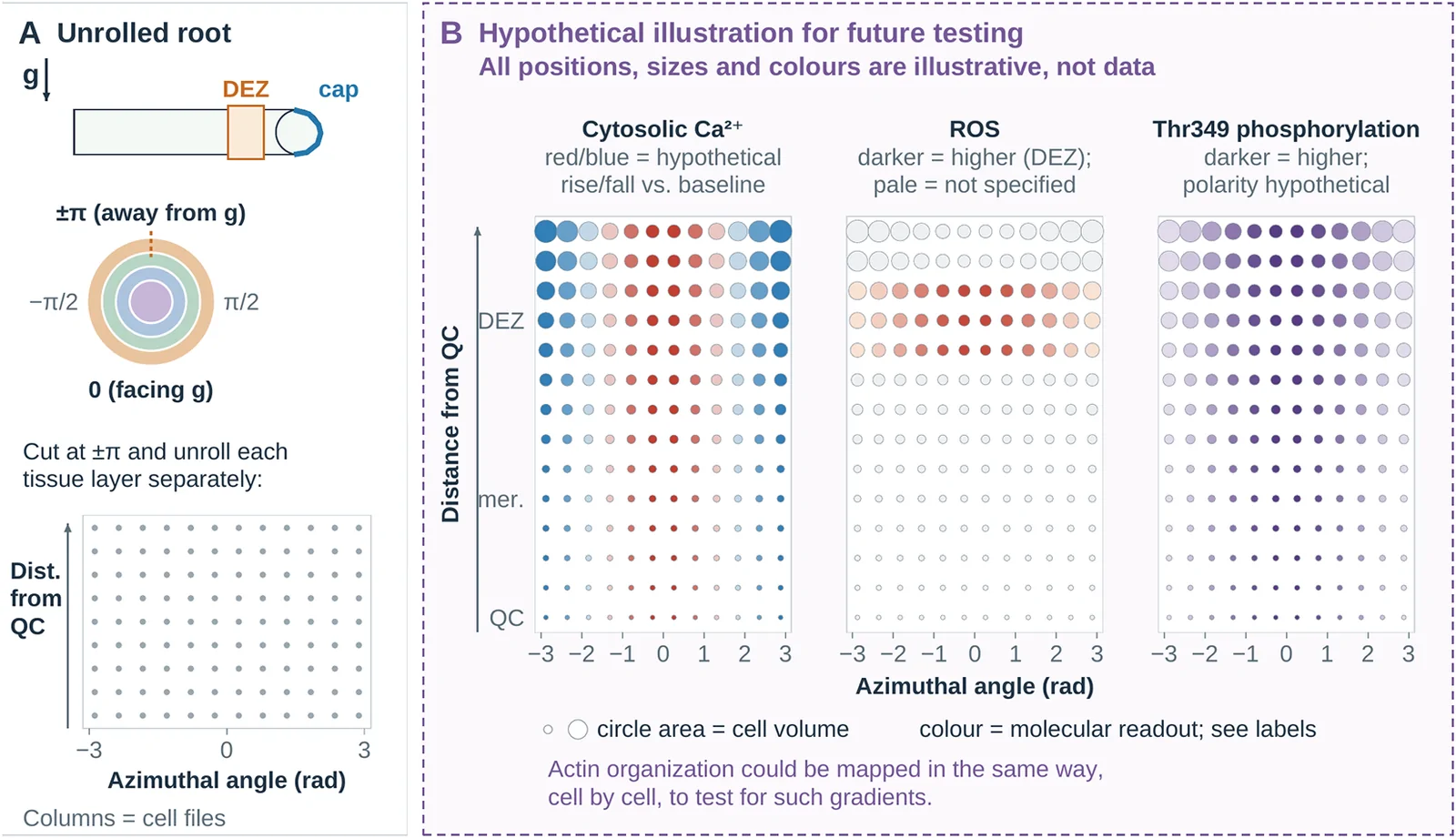
Unrolled root coordinates and a hypothetical illustration of cell-resolved responses to gravistimulation. **(A)** After reorientation of an Arabidopsis root, each cell can be assigned an azimuthal angle around the root axis relative to the gravity vector (g), from 0 on the side facing g to ±π on the side away from g, and a distance from the quiescent center (QC), as in the intrinsic root coordinate system (Schmidt et al., 2014). The root drawing marks the distal elongation zone (DEZ) and the root cap. Each tissue layer is cut at ±π and unrolled separately, so that cell files appear as columns and individual cells as circles. **(B)** Hypothetical illustration for future testing, in the same coordinates, of how cell volume (circle area) and three molecular readouts (color) could vary gradually among cell files of the root epidermis. The vertical axis is distance from the QC, with the meristem (mer.) and the DEZ indicated. For cytosolic Ca^2+^, red and blue indicate a hypothetical increase and decrease relative to the pre-reorientation baseline. For reactive oxygen species and α-tubulin Thr349 phosphorylation, darker color indicates a hypothetically higher level. Reactive oxygen species are illustrated only in the DEZ; pale grey circles elsewhere indicate that levels are not specified, not that reactive oxygen species are absent. All plotted positions, sizes, colors and gradients are illustrative and are not data. Published studies have compared only the side facing g and the side away from g: cytosolic Ca^2+^ in the root epidermis increased on the side facing g and decreased on the side away from g within minutes of a 180° rotation (Monshausen et al., 2011), reactive oxygen species were higher on the side facing g in the DEZ epidermis 1 to 2 h after reorientation (Krieger et al., 2016), and epidermal cells expand more on the side away from g during downward bending (Swarup et al., 2005). The polarities shown for Ca^2+^, reactive oxygen species and cell volume follow these two-sided comparisons; the polarity shown for Thr349 phosphorylation is hypothetical, because no spatial data are available. Actin organization could be mapped in the same way (Section 7).

## 4 Genetic dissection of α- and β-tubulin function in plant gravity response

### 4.1 The Arabidopsis tubulin gene families

The Arabidopsis genome contains six α-tubulin genes, *TUA1*–*TUA6*, encoding four distinct proteins: *TUA2* and *TUA4* encode the same protein, *TUA3* and *TUA5* encode the same protein, and *TUA1* and *TUA6* each encode a unique isoform (Kopczak et al., 1992). The Arabidopsis genome also contains nine β-tubulin genes, *TUB1*–*TUB9*, encoding eight isoforms, with *TUB2* and *TUB3* encoding the same protein (Snustad et al., 1992). Expression patterns differ among isoforms: *TUA1* is enriched in pollen, whereas *TUA6* is broadly expressed in vegetative tissues, and mutant phenotypes are not fully redundant, indicating that isoforms contribute differentially despite high sequence similarity. The partial non-redundancy becomes central to the “isoform paradox” (Section 7).

### 4.2 α-tubulin loss-of-function evidence

Antisense reduction of α-tubulin transcripts affects root growth, morphology, root-hair development, and root gravitropism in Arabidopsis (Bao et al., 2001). Gravitropic defects were observed in plants with otherwise moderate growth defects, suggesting that α-tubulin function is particularly important for directional growth and tissue organization. These findings established α-tubulin as a functional contributor to root gravitropism and raised the question of whether individual tubulin isoforms and specific tubulin residues make distinct contributions to directional growth.

### 4.3 Dominant tubulin mutants and structural insight

A forward-genetic screen recovered 32 Arabidopsis twisting mutants carrying missense mutations or amino-acid deletions in α- or β-tubulin (Ishida et al., 2007). The mutations mapped to three structural classes: the GTPase-activating region of α-tubulin, intra- and inter-dimer interfaces of the heterodimer, and lateral protofilament contacts. These dominant-negative tubulins incorporate into polymers and generate shallow helical cortical arrays whose handedness correlates with the handedness of organ twisting. GTPase-region mutants produce hyper-stabilized microtubules that can be decorated along their length by the otherwise plus-end-localized End Binding 1 protein, whereas intradimer-interface mutants produce more dynamic microtubules and distinct helical arrays (Ishida et al., 2007). These results show that specific tubulin residues control cortical array geometry and therefore organ-level directional growth, establishing, importantly, that single-residue changes in tubulin are sufficient to redirect growth, which is the structural premise behind treating a single phosphorylation site as functionally consequential.

### 4.4 *tua* mutants under altered gravity

Three Arabidopsis α-tubulin mutants, *tua3*, *tua4*, and *tua6*, have been examined at 1 g and 300 g hypergravity (Matsumoto et al., 2010). At 1 g, mutant hypocotyls were shorter and thicker than wild type and showed left- or right-handed helical growth. Hypergravity intensifies twisting, especially in *tua6*. The proportion of cells with longitudinal cortical microtubules was elevated in these mutants at 1 g and increases further under hypergravity. Gadolinium suppressed the hypergravity-induced increase in hypocotyl twisting in the tubulin mutants and inhibited the transverse-to-longitudinal reorientation of cortical microtubules in wild-type hypocotyls; lanthanum produced similar effects on hypocotyl growth and epidermal cell-file alignment. Among the three mutants, *tua6* showed the strongest hypocotyl twisting and the greatest shift of cortical microtubules toward an oblique or longitudinal orientation (**Table 2**). This phenotype was consistent with the strong transcriptional response of *TUA6* to hypergravity, although the relationship remains correlative (Matsumoto et al., 2007). Spaceflight extended the genetic evidence: in the Kibo module aboard ISS, inflorescence elongation was stimulated in both Arabidopsis wild-type Columbia and *tua6* under microgravity, but more so in *tua6*, and cell-wall extensibility increased in the mutant as well (Hoson et al., 2014). The developmental consequences of altered *TUA6* function are therefore gravity-dependent, and removing gravity can partially relieve constraints associated with the mutant phenotype.

**Table 2:** Tubulin-related mutants and lines with gravity-relevant phenotypes or mechanisms.

Mutant/line	Genetic basis	Approach	Gravity-related phenotype or mechanism	Reference
α-tubulin antisense lines	Reduced α-tubulin transcript	Transgenic suppression	Root gravitropism defect with altered root growth and root-hair development	(Bao et al., 2001)
*tua3*, *tua4*, *tua6* point/deletion alleles	EMS missense or deletion in α-tubulin	Forward screen; hypergravity assay	Helical growth at 1 g; twisting intensified at 300 g; *tua6* strongest	(Ishida et al., 2007; Matsumoto et al., 2010)
32 twisting mutants (α/β)	Mutations at GTPase region, dimer interfaces, lateral contacts	Forward genetic screen	Dominant-negative helical growth; array handedness correlates with twisting	(Ishida et al., 2007)
*tua6* in microgravity	Point mutation in *TUA6*	ISS spaceflight	Inflorescence growth stimulated 10-45%; stronger than WT	(Hoson et al., 2014)
GFP-TUA6 Thr349D/Thr349E	Phospho-mimic substitution at α-tubulin Thr349	Transgenic expression; live imaging	Phospho-mimic tubulin poorly incorporated into arrays	(Ban et al., 2013; Fujita et al., 2013)
*OsTUA* Thr349A	Ala substitution of the phosphosite	Phosphoproteomics; mutagenesis in rice cells	Thr349A abolishes stress-induced α-tubulin phosphorylation	(Ban et al., 2013)
*phs1* loss-of-function	Allele disrupting *PHS1*	Reverse genetics	Fails to depolymerize cortical microtubules and to accumulate phospho-α-tubulin under stress	(Fujita et al., 2013)

### 4.5 β-tubulin contributions

β-tubulin has been emphasized less than α-tubulin in gravity studies, but both subunits contribute to gravity-related cortical microtubule orientation. Hypergravity upregulates many α- and β-tubulin genes in Arabidopsis hypocotyls (Matsumoto et al., 2007), and *AtTUB2* and *AtTUB3*, which encode the same β-tubulin isoform, are among the tubulin genes upregulated under simulated microgravity in roots (Yemets et al., 2024). Genetic evidence further supports a role for β-tubulin, as mutations in either α- or β-tubulin can produce helical cortical microtubule organization and twisting growth (Ishida et al., 2007). Results from both α- and β-tubulin mutants indicate that each subunit contributes to cortical microtubule organization and directional growth, arguing against interpretation of the cytoskeletal response as an α-tubulin-specific phenomenon.

## 5 Regulatory layers shaping microtubule behavior

### 5.1 Transcriptional regulation under altered gravity

Gravity loading modulates transcript abundance for tubulin and microtubule-regulator genes in several systems. In azuki bean epicotyls, hypergravity transiently increases γ-tubulin complex genes *VaTUG* and *VaGCP3* (Soga et al., 2008) and katanin p60 *VaKTN1* (Soga et al., 2009); these responses are rapid, transient, and blocked by lanthanum or gadolinium. Hypergravity increases the expression of tubulin genes in Arabidopsis hypocotyls. All six α-tubulin genes (*TUA1*-*TUA6*) are upregulated, although the magnitude of the response differs among isoforms (Saito et al., 2003). Most β-tubulin genes also show increased expression under hypergravity (Matsumoto et al., 2007). Under simulated microgravity, *AtTUA2*, *AtTUA3*, *AtTUB2*, and *AtTUB3* are upregulated alongside *ATG8* genes in roots (Yemets et al., 2024). In true spaceflight, however, many tubulin genes were downregulated under microgravity in apical inflorescence tissue (Kato et al., 2022). These differences probably reflect differences in tissue, developmental stage, platform, or time scale, and they strongly caution against treating altered gravity as a single transcriptional state. They also caution against reading any one spaceflight transcriptome as definitive, since environmental variables such as light interact substantially with the spaceflight transcriptomic response. The same caution applies within a single dataset: in BRIC-20 seedlings, several α- and β-tubulin genes (including *TUA4*, *TUA6*, *TUB1*, and *TUB5*) are up-regulated under spaceflight, opposite to the downregulation reported in inflorescence apices (Kato et al., 2022), and the autophagy genes *ATG8b*, *ATG8e*, and *ATG8f* are down-regulated, contrary to their up-regulation under root clinorotation (Yemets et al., 2024).

### 5.2 γ-tubulin nucleation and katanin severing

Cortical microtubule orientation is continuously remodeled by nucleation, polymer dynamics, severing, and selective stabilization. γ-Tubulin ring complexes are the principal microtubule nucleators across eukaryotes (Kollman et al., 2011). In plant cortical arrays, γ-tubulin complexes are recruited to existing cortical microtubules, where they nucleate new microtubules at characteristic angles and contribute to self-organization (Murata et al., 2005); the augmin complex provides microtubule-dependent nucleation in interphase cells (Liu et al., 2014). Katanin severing complements nucleation by generating new ends and by amplifying new orientations during reorientation. The altered-gravity evidence for these activities is strongest at the transcript level, particularly for γ-tubulin complex genes and katanin in azuki bean epicotyls (Soga et al., 2008, 2009). Direct measurements of γ-tubulin recruitment, nucleation rate, and katanin severing activity during defined gravity responses remain limited and should be prioritized; recent structural work on plant augmin (Ashaduzzaman et al., 2025) further sharpens the mechanistic questions that such measurements could now address.

### 5.3 Post-translational regulation of α-tubulin: Thr349 phosphorylation

The most mechanistically characterized post-translational regulation of plant α-tubulin is phosphorylation of threonine 349. The proximal kinase is PROPYZAMIDE-HYPERSENSITIVE 1 (PHS1), first identified through a screen for propyzamide hypersensitivity (Naoi and Hashimoto, 2004). The semi-dominant phs1–1 allele carries an Arg-to-Cys substitution in a conserved non-catalytic N-terminal region of a MAPK phosphatase-like protein and compromises cortical microtubule organization. PHS1 is unusual in containing both a MAPK phosphatase-like domain and an atypical kinase domain with homology to slime-mold actin-fragmin kinase. Expression of phosphatase-inactive PHS1 caused seedling dwarfism, radial expansion of root epidermal cells, and extensive cortical microtubule depolymerization (Fujita et al., 2013). A central PHS1 fragment lacking the phosphatase domain was sufficient to destabilize microtubules, indicating that the phosphatase domain normally suppresses the intrinsic tubulin kinase activity of PHS1. Recombinant PHS1 showed Mn^2+^ dependent kinase activity, and mass spectrometry identified Thr349 of α-tubulin as a PHS1-dependent phosphorylation site (Fujita et al., 2013).

Independent support for Thr349 as the stress-responsive site came from phosphoproteomic analysis of hyperosmotically stressed rice culture cells, followed by alanine substitution of conserved serine and threonine residues (Ban et al., 2013). Among the mutants, Thr349A abolished hyperosmotic-stress-induced α-tubulin phosphorylation, the expected result if Thr349 is the modified residue, since alanine cannot be phosphorylated. The response was rapid, appearing within ~2 min of hyperosmotic treatment, and reversible after stress removal (Ban et al., 2013). Propyzamide, oryzalin, and both high- and low-temperature stress also induced α-tubulin phosphorylation, whereas Taxol partially suppressed hyperosmotic-stress-induced phosphorylation (Ban et al., 2013). The PHS1-Thr349 module thus integrates several distinct stress inputs onto a single tubulin residue, a property central to its candidacy as an integration point.

### 5.4 Structural and functional consequences of Thr349 phosphorylation

Thr349 lies at the longitudinal interface between adjacent αβ-tubulin heterodimers within a protofilament (Nogales et al., 1999). Phosphorylation at this position introduces a negative charge that is expected to interfere with longitudinal tubulin interactions and reduce microtubule polymerization. Phospho-mimic substitutions support the prediction: GFP-TUA6 carrying Thr349D failed to incorporate efficiently into mCherry-β-tubulin-labelled microtubules in Arabidopsis pavement cells, and an equivalent Thr349E substitution also impaired incorporation (Ban et al., 2013; Fujita et al., 2013). Tubulin purified from plants expressing induced PHS1ΔP-GFP failed to assemble efficiently, even with Taxol, *in vitro*, whereas phs1 loss-of-function material failed to show normal stress-induced cortical microtubule depolymerization or phospho-α-tubulin accumulation (Fujita et al., 2013). Together, these data demonstrated that PHS1-mediated Thr349 phosphorylation produced a polymerization-incompetent or polymerization-impaired α-tubulin pool.

A striking feature is the apparent effectiveness of Thr349 phosphorylation at sub-stoichiometric levels: relatively little phospho-α-tubulin can destabilize cortical microtubule reorientation, implying a dominant-negative mechanism. One plausible model is that phospho-tubulin dimers transiently engage growing plus ends and destabilize the GTP cap, promoting catastrophe. The model is consistent with biochemical reasoning from polymerization-blocked yeast tubulin mutants (Johnson et al., 2011), but the precise biophysical mechanism has not been demonstrated directly in plant microtubules, another point at which the current argument rests on non-plant tubulin and which therefore defines a concrete experimental target (Section 7).

### 5.5 Apparent plant-lineage specificity of the PHS1-Thr349 switch

Thr349 is conserved across many eukaryotic α-tubulins, including fungi and animals, but stress-inducible phosphorylation of the residue is currently best supported in plants and green algae. Fujita et al. (2013) noted that Thr349 phosphorylation was not reported in major animal phosphoproteomic databases available at the time (Fujita et al., 2013). Work in Chlamydomonas indicated that PHS1-dependent Thr349 phosphorylation also occurs in a green-algal lineage under hyperosmotic stress, and that PHS1 genes are distributed across freshwater Chlorophyta while being absent from some marine algae (Ng et al., 2022). The residue is therefore broadly conserved, but the PHS1-based, stress-responsive phosphorylation mechanism appears to be a plant/green-lineage regulatory solution rather than a universal eukaryotic tubulin switch. Experimental evidence for Thr349 phosphorylation (Ban et al., 2013; Fujita et al., 2013) has so far been obtained from only a limited range of plant species and experimental systems. Broader sampling is needed to determine whether the observed phosphorylation pattern is conserved across plant lineages or restricted to particular groups.

### 5.6 Other tubulin modifications and additional kinases

Thr349 phosphorylation does not exhaust the post-translational regulation of plant tubulins. β-tubulin can be phosphorylated by casein kinase 1-like 6 (Ben-Nissan et al., 2008) and by NIMA-related kinases (Motose et al., 2011), with consequences for cortical array organization. Plant tubulins also undergo other post-translational modifications, including acetylation (Nakagawa et al., 2013), detyrosination/tyrosination and nitrosative-stress-related modifications (Jovanović et al., 2010), contributing to a broader “tubulin code” (Gardiner, 2019). It is not yet known whether Thr349 phosphorylation interacts with other tubulin modifications. For example, α-tubulin acetylation may alter its effect on polymerization.

## 6 Integrating cortical microtubule regulation into plant gravity signaling

### 6.1 Three regulatory layers

Current evidence indicates that cortical microtubule behavior during gravity responses is controlled through at least three distinct regulatory layers (**Table 3**). 1) A transcriptional layer adjusts the tubulin pool and the abundance of nucleation and severing machinery over hours to days. 2) A regulator-mediated layer controls existing arrays through γ-tubulin-dependent nucleation, katanin severing, and microtubule-associated proteins over minutes to hours. 3) A post-translational layer modifies tubulin itself, with PHS1-mediated Thr349 phosphorylation acting within seconds to minutes. These layers are not redundant: they operate on different time scales, affect different features of array behavior, and probably interact during sustained responses. The post-translational modifications can provide rapid adjustment, regulator-mediated control can remodel existing arrays, and transcriptional regulation can reshape cellular capacity for longer-term adaptation. Framing the cytoskeletal response as layered, rather than as a single mechanism, is the organizing claim of the present review. Support for the layers being separable, at the whole-seedling level, comes from spaceflight seedlings, in which reduced α-tubulin Thr349 phosphorylation and increased CELLULOSE SYNTHASE 1 phosphorylation occur without matching changes in the corresponding transcripts (GLDS-38) (Kruse et al., 2020), showing the fast post-translational layer operating independently of the slow transcriptional one. Because these are whole-seedling measurements, they do not establish that the layers are uncoupled within individual cells.

**Table 3:** Regulatory layers controlling cortical microtubule behavior during plant gravity response.

Layer	Time scale	Key components	Evidence related to altered gravity	References
Transcriptional	Hours-days	α/β-tubulin, γ-tubulin complex, katanin, ATG8 genes	Hypergravity upregulates tubulin/γ-tubulin/katanin transcripts in some systems; simulated microgravity alters tubulin and ATG8; true spaceflight can downregulate tubulin genes in specific tissues	(Matsumoto et al., 2007; Soga et al., 2008, 2009; Kato et al., 2022; Yemets et al., 2024)
Regulator-mediated	Minutes-hours	γ-TuRC; augmin; katanin; MAP65 and other MAPs	Core regulators organize cortical arrays; gravity-specific activity data are limited, but altered-gravity transcripts implicate nucleation and severing	(Murata et al., 2005; Soga et al., 2008, 2009; Liu et al., 2014)
Post-translational	Seconds-minutes	α-tubulin Thr349 phosphorylation by PHS1; β-tubulin phosphorylation by CK1-like and NIMA-related kinases	Thr349 phosphorylation reduced under spaceflight conditions across several α-tubulin peptides; same site responds rapidly to osmotic, salt, temperature and drug stresses	(Ban et al., 2013; Fujita et al., 2013; Kruse et al., 2020)

### 6.2 Thr349 phosphorylation as a candidate regulatory point: predictions and experimental outcomes that would challenge the model

Post-translational modification of α-tubulin at Thr349 occupies a distinctive position among these layers: it has a defined kinase, a known residue, a (homology-based) structural rationale, rapid and reversible kinetics, and spaceflight evidence for altered abundance (Ban et al., 2013; Fujita et al., 2013; Kruse et al., 2020). Three features make it an attractive candidate for a convergence point. First, several stress inputs, including hyperosmotic, salt, temperature, and microtubule-destabilizing drugs, converge on the same modification (Ban et al., 2013). Second, the modification has a direct effect on polymerization and a likely indirect effect on cellulose deposition through the microtubule-cellulose axis. Third, Thr349 phosphorylation is reduced across several Arabidopsis α-tubulin peptides under spaceflight conditions, with similar effect sizes in the BRIC-20/GLDS-38 dataset (Kruse et al., 2020).

The proposed model generates specific predictions that can be tested experimentally and compared with outcomes that would support or challenge it. If PHS1-Thr349 is a candidate regulatory mechanism in gravity response, then: (i) gravistimulation or a change in mechanical loading should alter Thr349 phosphorylation within minutes, ahead of array reorientation, in the cells where reorientation occurs and potentially with opposite sign on the upper and lower flanks; (ii) phospho-null (Thr349A) and phospho-mimic (Thr349D/E) alleles should blunt or distort, respectively, the cortical-array response to altered gravity, and should do so in the cell types where the response occurs; (iii) loss of PHS1 function should attenuate the rapid component of array remodeling under gravistimulation without abolishing the slower, transcriptionally driven component; and (iv) the spaceflight reduction in Thr349 phosphorylation should track a measurable shift toward more stable arrays in the same tissue. The hypothesis is falsified if Thr349 phosphorylation does not change on the time scale of array reorientation in the responding cells, if phospho-site alleles leave the gravity response intact, or if PHS1 loss has no effect on the rapid phase.

### 6.3 Alternative and complementary nodes

Thr349 phosphorylation is unlikely to be the only regulatory mechanism involved. γ-Tubulin-dependent nucleation helps establish new cortical microtubule orientations and thereby reshapes the overall microtubule pattern (Soga et al., 2008). Katanin severing can amplify new orientations by releasing fragments and generating new ends, and katanin transcripts respond to altered gravity in azuki bean epicotyls (Soga et al., 2009). Microtubule-associated proteins, including MAP65-family bundlers, are regulated by phosphorylation in other contexts and could provide further integration points. The current evidence supports a layered model with multiple convergence points operating with different timing, strength, and tissue specificity.

### 6.4 Connecting the cytoskeletal regulation to the auxin-mediated pathway and broader signaling

The gravitropic-signaling schematic of Wyatt and Kiss (2013) already includes phosphorylation/dephosphorylation and the actin cytoskeleton among its transduction components, but not the microtubule cytoskeleton; the nitric oxide- and reactive oxygen species-centered schematic provides a useful scaffold for canonical components (Kruse and Wyatt, 2022). The layered model proposed here specifies the phosphorylation component as the PHS1-Thr349 system and adds the microtubule regulatory pathway absent from these schematics. Crucially, the cytoskeletal regulatory pathway must be placed alongside the now well-resolved auxin-mediated pathway: amyloplast sedimentation repolarizes LAZY proteins to direct PIN-dependent auxin asymmetry (Chen et al., 2023), and the auxin branch is the dominant explanation for differential growth in gravitropism. The cytoskeletal regulatory pathway is not proposed to replace it. Rather, mechanical stress, calcium influx, reactive oxygen species, and MAPK pathways can be placed upstream of microtubule regulation; PHS1-Thr349 provides one rapid route from stress signaling to microtubule destabilization; and γ-tubulin, augmin, katanin, and MAPs provide routes for array remodeling. Downstream, array organization influences cellulose synthase trajectories and wall anisotropy and may feedback on auxin transport through changes in cell shape, membrane organization, or trafficking. Consistent with treating the two arms together rather than separately, the same spaceflight seedling dataset shows significant transcript-level upregulation of *ATPIN3* and *AtLAZY1* alongside cytoskeletal changes (GLDS-38) (Kruse et al., 2020). Direct links from nitric oxide or from the LAZY/PIN axis to PHS1 remain uncharacterized and should be presented as research opportunities rather than established pathways (**Figure 1**).

## 7 Discussion and future directions

What activates PHS1 during gravity response? PHS1 activation under osmotic stress is better supported than activation during gravity response. It remains unknown whether mechanical stress, statolith-dependent signaling, calcium influx, reactive oxygen species, or MAPK cascades directly activate PHS1 during gravistimulation. Experiments should compare true reorientation, hypergravity, and mechanical indentation or bending while measuring Thr349 phosphorylation within minutes. Because responses can be local and of opposite sign between flanks and cell types (Section 3.5), these measurements should be made *in situ* with cellular and positional resolution rather than in whole-organ extracts (Pasternak and Yaroshko, 2026).

What phosphatase reverses Thr349 phosphorylation? The rapid loss of phospho-α-tubulin after stress removal implies active dephosphorylation, but the responsible phosphatase has not yet been identified. PP2A- and PP1-type phosphatases are candidates, but direct biochemical and genetic evidence is needed. Identifying the eraser is as important as identifying the writer for establishing a dynamic, signal-responsive switch.

How is the isoform paradox resolved? Spaceflight phosphoproteomic data report reduced Thr349 phosphorylation across TUA1, TUA3, TUA4, and TUA6 with similar magnitudes (Kruse et al., 2020), yet single-isoform mutants show distinct phenotypes (Bao et al., 2001; Ishida et al., 2007; Matsumoto et al., 2010; Hoson et al., 2014). If Thr349 modification operates broadly across isoforms, isoform-specific phenotypes probably arise from expression domain, developmental timing, subcellular localization, differential incorporation, or distinct partners; isoform-resolved imaging and reporter lines could settle these alternatives. The paradox is compounded by a measurement limit: because TUA1, TUA4, and TUA6 share the Thr349 tryptic peptide, current phosphoproteomics cannot assign the modification to a specific isoform among them, so isoform-resolved tools are needed to define the phenotype, not only to explain it.

In which cell types does Thr349 phosphorylation operate? Gravitropism and gravity resistance involve different cell populations. Thr349 phosphorylation could act in statocytes responding to amyloplast sedimentation, in elongation-zone cells experiencing differential growth and mechanical stress, in non-statocyte tissues involved in gravity resistance, or across several cell types. Tissue-specific reporters and inducible phospho-null or phospho-mimic alleles would address the question directly and are prerequisites for prediction (ii) in Section 6.2. Microtubule involvement itself may be tissue-specific, because differential growth of maize root cortical cells during gravitropism was reported to be independent of the microtubular cytoskeleton (Baluška et al., 1996). To our knowledge, the direction and timing of microtubule responses to gravistimulation have not been compared directly between cortical and vascular cells of Arabidopsis roots. Current whole-seedling phosphoproteomic data also cannot indicate where changes in Thr349 phosphorylation occur.

Most spatial data on gravity responses compare only the upper and lower flanks. After gravistimulation, however, the root is symmetric only about the vertical plane containing the gravity vector, so each half behaves as a semi-cylinder in which responses may change progressively with azimuthal angle from the lower to the upper side, rather than forming discrete upper and lower halves. Three-dimensional cellular maps that assign each cell a position by azimuthal angle and distance from the quiescent center, as provided by the intrinsic root coordinate system (Schmidt et al., 2014), could be used to quantify cell volume, Thr349 phosphorylation, reactive oxygen species, Ca^2+^, and microtubule and actin organization cell by cell in gravistimulated roots (**Figure 2B**). Few studies have yet taken this approach to gravity responses, and it would test whether these readouts vary as gradients among cell files according to azimuthal angle.

What is the dominant-negative biophysical mechanism? Sub-stoichiometric phospho-α-tubulin destabilizes arrays, but the mechanism in plant microtubules is unresolved. *In vitro* reconstitution with purified plant tubulin or engineered phospho-mimic/null tubulins could test whether phospho-tubulin blocks elongation, promotes catastrophe, alters rescue, or affects lattice repair, and would remove the current dependence on yeast-tubulin reasoning.

How do the layers interact? The three layers act on different time scales, but their hierarchy is unknown. One possibility is that rapid Thr349 phosphorylation initiates disassembly, followed by katanin- and γ-tubulin-dependent rebuilding and later transcriptional adjustment; another is that each layer responds independently to distinct upstream signals. Time-course experiments measuring Thr349 phosphorylation, microtubule dynamics, nucleation, severing, tubulin transcripts, and growth output in the same material would be especially informative.

How broadly conserved is the mechanism? Most mechanistic work is in Arabidopsis, rice, and Chlamydomonas. Conservation of Thr349 predicts structural importance across eukaryotes, but conservation of the PHS1-based stress-response mechanism is more restricted (Ng et al., 2022). Comparative studies across bryophytes, gymnosperms, crop monocots, and diverse algae would clarify when the PHS1-Thr349 module arose and how it diversified.

## 8 Conclusion

Plant gravity responses require coordination between gravity perception, intracellular signaling, cytoskeletal regulation, cell-wall organization, and differential growth, and the coordination differs between cell types and between cells of the same type at different positions relative to the gravity vector. Evidence from gravistimulation, hypergravity, simulated microgravity, spaceflight, and tubulin mutants shows that cortical microtubules participate in both gravitropism and gravity resistance. Their behavior is regulated at several levels, including changes in tubulin and regulator expression, γ-tubulin-dependent nucleation, katanin-mediated severing, and post-translational modification of tubulin.

PHS1-mediated phosphorylation of α-tubulin at Thr349 is currently the best-characterized post-translational mechanism affecting plant microtubule polymerization. Its rapid induction during stress and the reduced abundance of Thr349-containing phosphopeptides under spaceflight conditions make it a strong candidate for further investigation in gravity-related responses. However, direct activation of PHS1 during gravistimulation has not been demonstrated, the available phosphoproteomic data are whole-seedling averages, and changes in Thr349 phosphorylation may be local and of opposite sign in different cell types or flanks. The effects of altered Thr349 phosphorylation on cortical microtubule dynamics under spaceflight conditions also remain unknown. Thr349 phosphorylation should therefore be considered as one component of a broader regulatory system.

Auxin redistribution through the LAZY-PIN pathway remains the best-established mechanism driving differential growth during gravitropism. Cortical microtubule regulation is likely to act alongside and downstream of auxin signaling by controlling cellulose synthase trajectories, cell-wall anisotropy, and directional cell expansion. Future studies should combine Thr349 phospho-null and phospho-mimic mutants, defined *phs1* backgrounds, tissue-specific manipulation, and live imaging during gravistimulation with cellular and positional resolution. Simultaneous measurement of phosphorylation, microtubule dynamics, nucleation, cellulose deposition, and growth will be essential for determining how cytoskeletal regulation contributes to plant responses under different gravitational conditions.
